# Variability in arteriolosclerosis in different brain regions: An AI‐based population study of the Oldest‐Old (Vantaa85+)

**DOI:** 10.1002/alz70861_108734

**Published:** 2025-12-23

**Authors:** Kia Colangelo, Mikko I. Mäyränpää, Jarno Tuimala, Ville Kivistö, Eloise H Kok, Olli Tynninen, Henri Puttonen, Tuomo Polvikoski, Liisa Myllykangas

**Affiliations:** ^1^ University of Helsinki, Helsinki Finland; ^2^ HUS Diagnostic Center, Helsinki Finland; ^3^ Finnish Tax Administration, Helsinki, Uusimaa Finland; ^4^ University of Helsinki, 00290 Helsinki, Uusimaa Finland; ^5^ Tampere University Hospital (TAYS), Tampere, Pirkanmaa Finland; ^6^ Wellbeing Services County of Pirkanmaa, Tampere, Pirkanmaa Finland; ^7^ Tampere University, Tampere, Pirkanmaa Finland; ^8^ University of Helsinki, Helsinki, Uusimaa Finland; ^9^ HUS Diagnostic Center, Helsinki, Uusimaa Finland; ^10^ Newcastle University, Newcastle upon Tyne UK

## Abstract

**Background:**

Brain arteriolosclerosis, characterized by the thickening of arteriolar walls, is frequently observed in autopsy studies of the oldest‐old and associated with cognitive decline. We aimed to develop an AI–based algorithm for quantifying arteriolar wall thickness, and to investigate potential associations between arteriolosclerosis and vascular risk factors, cognitive outcomes, and coexisting neuropathological comorbidities. The level of arteriolosclerosis can be studied by defining Sclerotic index (SI) which calculates external and internal luminal diameter.

**Method:**

The Vantaa 85+ cohort, comprising 601 residents of Vantaa, Finland, aged ≥85 years in 1991. and underwent cognitive assessments, were utilized in this study. Neuropathological examinations were completed for 304 participants Hematoxylin‐ and eosin‐stained sections from frontal cortex (FC), hippocampus (HC) and basal ganglia (BG) were used to train the AI model. The algorithm was trained to identify tissue, vessel wall, and lumen, as well as white and gray matter within the FC. The algorithm measured all vessels, and those sized 20‐120μm were used in the analyses. The model was validated by three independent pathologists. With arteriolar wall thickness we calculated SI for each vessel using R.

**Result:**

In this very elderly population, the level of arteriolosclerosis varied between brain regions, but the average SI was severe (SI>0.5) in all 3 areas studied (FC SI=0.62, BG SI=0.57, HC SI=0.55). In HC, arteriolosclerosis was significantly associated with LATE (*p*=0.011), Braak NFT stage (*p*=0.046), and the TMEM‐106B variant (*rs1990622*) (*p*=0.012). Arteriolosclerosis in FC was not associated with any cognitive or pathological variables but was with the ABCC9 variant (*rs704178*) (*p*=0.013). Arteriolosclerosis in BG was significantly associated with large (*p* =0.004) and small brain infarcts (*p*=0.021), circle of Willis atherosclerosis (*p*=0.029), dementia (*p*=0.0013), last MMSE before death (*p*=0.004), and the ABCC9 variant (*p*=0.004). We did not find associations between arteriolosclerosis and most vessel disease risk factors such as smoking, diabetes or hypertension in any of the studied brain areas.

**Conclusion:**

Arteriolosclerosis severity, and associated cognitive outcomes and comorbidities varied across brain regions and most conventional vascular risk factors did not influence the degree of arteriolosclerosis in this population‐based study of the oldest‐old.

